# Novel regulatory SNPs that can be activated due to metformin treatment may orchestrate liver gluconeogenesis and add to the variability in AMPK-dependent mechanisms of metformin response

**DOI:** 10.18699/vjgb-26-29

**Published:** 2026-04

**Authors:** E.E. Korbolina, I.S. Damarov, T.I. Merkulova

**Affiliations:** Institute of Cytology and Genetics of the Siberian Branch of the Russian Academy of Sciences, Novosibirsk, Russia; Institute of Cytology and Genetics of the Siberian Branch of the Russian Academy of Sciences, Novosibirsk, Russia; Institute of Cytology and Genetics of the Siberian Branch of the Russian Academy of Sciences, Novosibirsk, Russia

**Keywords:** metformin, individual drug response, TD2M, regulatory SNPs, gluconeogenesis, AMPK-dependent mechanisms, signal transduction pathways (FoxO, TNF-α, TGF-β), метформин, индивидуальный ответ на препарат, сахарный диабет второго типа, регуляторные полиморфизмы (rSNPs), глюконеогенез, АМФК-зависимая киназа, FoxO, TNF-α, TGF-β-сигнальные пути

## Abstract

Metformin is a first-line therapy for type 2 diabetes, yet individual response varies significantly, with over 30 % of patients failing to achieve optimal glycemic control. The specific regulatory mechanisms of this phenomenon remain poorly understood and genetic variants involved are mainly undiscovered. There are multiple lines of evidence that the leading role in determining the variance in phenotypic outcome belongs to regulatory SNPs (rSNPs) as they directly modify gene expression. Therefore, the genome-wide search for such functional variants and deciphering associated phenotypes stands as a fundamental challenge. Previously, based on the results of bioinformatics analysis of allele-specific expression and binding landscape in human peripheral blood mononuclear cells, we have established an original panel of 14 796 rSNPs within promotors of 5132 genes. Aiming to pinpoint functional variants most likely linked to metformin hepatic response and impacts on liver gluconeogenesis, we analyzed the relevant open-access data as well as rSNPs from our panel and the corresponding genes. 1196 genes reported to be regulated by metformin in human hepatocytes and 115 genes involved in gluconeogenesis and/or its regulation via Gene Ontology annotations were intersected. Free R software and STRING v.11 tools were used for functional annotation. A number of genes harboring rSNPs within promotor regions were found to be particularly implicated in the mechanisms of metformin’s action. Functional enrichment analyses revealed enrichment in critical pathways including FoxO, TNF-α and TGF-β signaling, also implicated in diabetes complications. Among these, six genes (ARPP19, ATF4, NR3C1, PFKFB3, TCF7L2, and WDR5) were strongly associated with regulation of gluconeogenesis, and may be modulated by metformin in the liver. We conclude that metformin therapy response may be influenced by the newly identified functional SNPs including rSNPs within the promotors of genes for gluconeogenic enzymes and transcription regulators

## Abstract (ru)

Метформин является препаратом первого выбора для лечения сахарного диабета второго типа, однако более чем у 30 % пациентов не достигается оптимальный гликемический контроль. Лежащие в основе этого феномена регуляторные механизмы и ассоциированные генетические варианты остаются неизученными. Накопленные данные свидетельствуют о том, что ключевую роль в определении фенотипической вариабельности играют функциональные генетические варианты, в особенности регуляторные однонуклеотидные полиморфизмы (rSNP), непосредственно влияющие на уровень экспрессии генов. Полногеномный поиск таких функциональных вариантов и выявление их фенотипических последствий – актуальная научная задача. Ранее на основе результатов биоинформатического анализа аллель-специфической экспрессии и профиля распределения активирующих гистоновых модификаций в мононуклеарных клетках периферической крови человека нами была сформирована оригинальная панель из 14 796 rSNP, локализованных в промоторах 5132 генов. Целью настоящей работы был поиск в нашей панели rSNP вариантов, потенциально связанных с регуляцией процессов глюконеогенеза в печени, в том числе под действием монотерапии метформином, с помощью анализа независимых релевантных полногеномных данных. Анализировали 1196 генов, экспрессия которых в гепатоцитах человека изменялась под влиянием метформина АМФК-зависимым образом, а также 115 генов, вовлеченных в глюконеогенез и/или его регуляцию, согласно данным генных онтологий. Для функциональной аннотации использовали инструменты свободного программного обеспечения R и базы данных STRING (v.11). В результате анализа был выделен ряд генов, которые, вероятно, играют особую роль в механизмах действия метформина и индивидуальной чувствительности к препарату, в промоторных районах которых нами были картированы rSNP. Функциональный анализ обогащения выявил вовлеченность этих генов в ключевые сигнальные пути, ассоциированные также с осложнениями сахарного диабета второго типа. Для шести генов (ARPP19, ATF4, NR3C1, PFKFB3, TCF7L2 и WDR5) экспрессия не только тесно связана с регуляцией глюконеогенеза, но также может модулироваться в гепатоцитах под действием метформина. Мы предполагаем, что rSNP в промоторах генов ферментов, участвующих в глюконеогенезе, и транскрипционных факторов могут играть существенную роль в механизмах формирования индивидуального ответа на терапию метформином

## Introduction

Metformin, an oral biguanide agent, is the first-line medication to reduce blood glucose levels in patients with T2DM. Although its mechanism of action is still not fully understood, metformin has been shown to decrease liver gluconeogenesis and enhance insulin sensitivity in muscle and adipose tissue (Rena et al., 2017; LaMoia, Shulman, 2021). Moreover, its beneficial pleiotropic effects beyond glycemic control are well known, which include lowering the incidence of cardiovascular events (Bu et al., 2022), correcting energy metabolism in neurologic disorders (Loan et al., 2024), and regulating inflammatory markers (Karbalaee-Hasani et al., 2021).

At the molecular level, metformin can inhibit complex I of the mitochondrial electron transport chain in hepatocytes (El- Mir et al., 2000), leading to decreases in ATP and increases in AMP levels and in turn activating AMP-activated protein kinase (AMPK), a master controller of metabolic homeostasis (Smiles et al., 2024). The activation of AMPK results in suppression of gluconeogenic gene expression and reduction in glucose output (LaMoia, Shulman, 2021). Moreover, there is evidence that the metformin glucose-lowering effects in hepatocytes can occur via AMPK-independent mechanisms. In particular, increased AMP levels can directly suppress gluconeogenesis via allosteric inhibition of fructose-1,6-bisphosphatase 1, FBP1 (Hunter et al., 2018). There is also an increasing interest in examining the effects of AMPK activity modulation in chronic metabolic diseases and cancer (Strang et al., 2025), including using the activators and inhibitors with effects independent of AMPK, i. e. compound C, an ATP-competitive AMPK inhibitor (which was reported to disrupt various biological events).

However, metformin’s response varies among individuals. As is shown, metformin is ineffective in over 30 % of cases because of various factors, including genetic ones (Cook et al., 2007; St-Amour et al., 2025). Currently, the search for genetic variants (mainly SNPs) associated with drug response efficacy is mainly focused on the genes coding for phase I and II drug metabolizing enzymes, drug transporters, and a number of upstream transcription regulators (Gaedigk et al., 2020; Rykova et al., 2022). Metformin is not metabolized in the human body (Gong et al., 2012); correspondingly, most studies aimed to identify the SNPs associated with the response to metformin were focused on the genes coding for organic cation transporters (OCTs) and the multidrug and toxin extrusion (MATE) proteins. This allowed for detection of a number of SNPs in the genes of these groups (Zhou et al., 2015; Xiao et al., 2016). The use of unbiased genome-wide approach (GWAS) succeeded in finding several SNPs associated with the response to metformin in genes, the products of which are involved in other functions. These SNPs have been discovered in regulatory regions of the ATM gene, the product of which is involved in the maintenance of redox homeostasis in the cell (GoDARTS and UKPDS Diabetes Pharmacogenetics Study Group et al., 2011); the PRPF31 gene, coding for pre-mRNA processing factor 31; the CPA6 (carboxypeptidase A6) gene, associated with the regulation of the Akt/mTOR signaling pathway; and the STAT3 gene, coding for eponymous transcription factor, which is a well-known regulator of metabolic and immune processes (Rotroff et al., 2018).

Nevertheless, GWAS application in pharmacogenomics yet failed to provide a considerable progress, mainly because of the difficulties (and more often, impossibility) in forming large cohorts (tens and hundreds of thousands) of patients to determine the effects of each drug. Modern methods of functional genomics – eQTL analysis and detection of allele-specific events in omics data – do not suffer from this limitation (Degtyareva et al., 2021). However, the studies utilizing these approaches are few and have even absent at all for antidiabetic drugs, including metformin. Earlier, we measured the allelic imbalance at the level of gene expression and the profiles of active chromatin marks in the paired RNA-seq and ChIP-seq data for peripheral blood mononuclear cells (PBMCs) of nine healthy donors and constructed a panel of 14 796 regulatory SNPs (rSNPs) potentially able to influence the expression levels of the genes harboring these SNPs in their promoters (Damarov et al., 2024). Then, using the RNA-seq data (GSE153315, GEO DataSets) on the blood cells of T2DM non-responders to metformin (N = 10) and T2DM responders (N = 10) (Vohra et al., 2022), we found 367 rSNPs from our panel in the promoters of 131 corresponding differentially expressed genes (DEGs). Primary analysis of this gene set revealed a number of transcriptional regulators both known to be involved in T2DM pathogenesis and still poorly studied (Damarov et al., 2024). Here, we performed further analysis of the obtained rSNP list using independent transcriptomic data on metformin hepatic response (Luizon et al., 2016) and available data on metformin impacts on liver gluconeogenesis

## Materials and methods

Gene sets analyzed. Three original gene sets were compared, and first was the set of 5132 genes harboring the rSNPs in promotor regions based on our previous analysis of alleleasymmetric events in omics data (RNAseq and ChIP-seq) for human PBMCs of nine healthy donors (Damarov et al., 2024). Each rSNP in the reported panel was located within ± 1000 bp from known TSS (transcription start site) and associated with allele-asymmetric binding of activating histone modifications (H3K4me3 and/or H3K27ac, ChIP-seq). Moreover, when analyzing RNA-seq data obtained for the same PBMCs samples, allele-specific expression events were observed within the transcribed sequences of the corresponding genes. Based on the association of the identified regulatory variants with two types of allele-specific events, we assumed that our rSNPs were most likely affecting the expression of certain human genes.

The second gene set comprised 1196 AMPK-dependent genes that were identified through clustering analysis on all DEGs (padj < 0.05) identified from RNA-seq data across the three conditions (treated with metformin, metformin + an AMPK inhibitor (compound C) or no treatment for 8 hours in triplicate) of cultured primary human hepatocytes from a sole male donor (Luizon et al., 2016). The DEGs were termed AMPK-dependent when found differently expressed between hepatocytes with metformin treatment and controls; however, their expression was restored to the normal level (or similarly altered) in hepatocytes with the combined treatment of compound C and metformin. And the third gene set consisted of 115 genes that were classified as involved only in gluconeogenesis, only in regulation of gluconeogenesis, or shared both Gene ontology terms (Ashburner et al., 2000). The analyzed human datasets were under ethical consent agreements as stated in authorized submissions.

Functional annotation. To predict the biological processes and metabolic pathways associated with the analyzed gene lists, a search for associated terms from GO and KEGG gene ontologies and metabolic pathways (Kanehisa, 2000) was performed using the clusterProfiler package (v4.15.1) (Yu et al., 2012) when taking into account the Benjamini–Hochberg correction (p.adj < 0.1).

A functional protein association network (PPI) was built and analyzed by STRING v.11 tools (Szklarczyk et al., 2023). The k-means clustering was applied in STRING on the basis of evidence score and connection cutoff at 0.40; the enrichments of the KEGG and Wiki pathways were analyzed with a p-value cutoff of < 0.05 and minimum count in the network setting of 2.

## Results and discussion

**Fig. 1.**
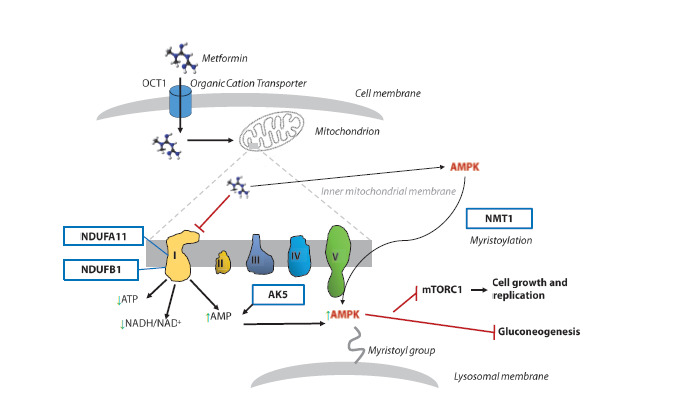
Antihyperglycemic effects of metformin are implemented through inhibition of complex I of mitochondrial electron transport chain and activation of AMP kinase. The proteins associated with the AMPK pathway are denoted with blue frames. Green vertical arrows indicate a decrease or an increase in the amount of metabolite or enzyme activity; blunt red arrows indicate inhibition; black arrows indicate membrane transport or a functional connectivity; the complexes of the electron transport chain embedded in the inner mitochondrial membrane are labeled with roman numerals (I through V); NDUFA11, NADH:ubiquinone oxidoreductase subunit A11; NDUFB1, NADH:ubiquinone oxidoreductase subunit B1; AK5, adenylate kinase 5; AMPK, AMP-activated protein kinase; NMT1, N-myristoyltransferase 1; and OCT1, organic cation/carnitine transporter 1.

Matching well the generally accepted model for metformin action, our panel of rSNPs located within the promotors of DEGs associated with metformin response (Damarov et al., 2024) included rSNPs within promotors of genes for key enzymes of mitochondrial electron transfer chain (NDUFA11 and NDUFB1) and upstream/downstream AMPK regulators (Fig. 1). So, AK5 adenylate kinase increases the phosphorylation of AMPK (Dzeja, Terzic, 2009); and N-myristoyltransferase (NMT1) controls the N-myristoylation and lysosomal localization of the AMPK protein (Chen et al., 2020). Activated AMPK inhibits the mTOR (mammalian target of rapamycin) pathway, a pivotal regulator of cell metabolism and growth (Gwinn, Shaw, 2010; Smiles et al., 2024).

**Fig. 2.**
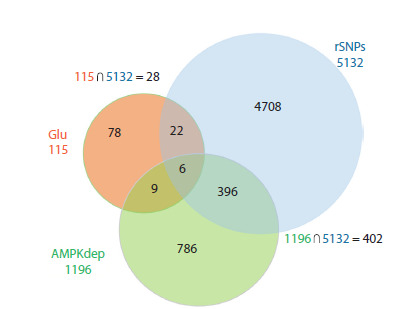
Venn diagram representing the intersection between the ‘Glu’ (orange), ‘rSNP’ (blue) and ‘AMPKdep’ (green) gene sets. ∩ indicates overlapping; the numerical values in the intersections show the counts for corresponding gene groups

To gain a better understanding of the possible involvement of our rSNPs in the mechanisms underlying the antihyperglycemyc effects of metformin treatment, we compared and analyzed three gene sets (Fig. 2). The ‘AMPKdep’ set of 1196 AMPK-dependent genes associated with metformin hepatic response was obtained from (Luizon et al., 2016), the ‘rSNPs’ set of 5132 genes with the regulatory variants originally identified within promoter regions was previously reported in (Damarov et al., 2024). Then, the ‘Glu’ set consisted of genes related to gluconeogenesis in GO terms (see the Materials and Methods section for details).

**Fig. 3.**
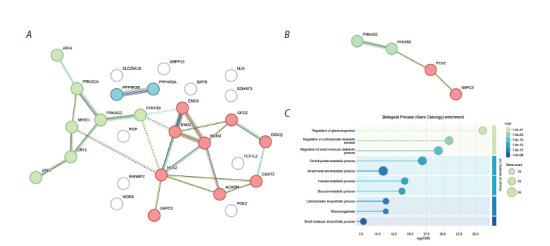
Interpreting a list of 402 genes with pathway enrichment analysis. (A) The most relevant enriched KEGG terms obtained using R (Materials and Methods). (B) Wiki pathway enrichment determined and visualized by STRING. ID, gene or pathway identifier from the database; FE, Fold Enrichment; p.Adjust, a p-value as corrected with the Benjamini–Hochberg procedure; count, the number of genes presented in the list; and ER, endoplasmic reticulum (A). Different colors indicate top enriched terms with corresponding p-values (FDR) according to the color panel (B).

Combining the results of our previous analysis with the AMPKdep gene set allowed to identify 402 genes, the expression of which in hepatocytes may be influenced by our rSNPs. The results of functional enrichment analysis using R signified that Protein processing in endoplasmic reticulum, Apoptosis and FoxO signaling KEGG pathways were enriched in a given set (Fig. 3A). Using the enrichment analysis in STRING, we observed the enrichment of the same gene list belonging to several Wiki metabolic pathways: similarly, apoptosis and, in addition, adipogenesis and signaling pathways including TNF-alpha-, TGF-beta- and angiogenesis-related VEGFA\ VEGFR2-signaling (Fig. 3B).

It is worth to mention that the signaling pathways above were widely reported to be affected by metformin (Yi et al., 2016; Kristófi, Eriksson, 2021; Pan et al., 2024). For example, some mice data indicate the Foxo1 protein to be one of the key targets of metformin in regulating blood glucose and hepatic glucose production under therapeutic concentrations (Guo et al., 2021). Moreover, the same pathways are commonly known as mediators that orchestrate and propagate inflammatory responses and thereby play a crucial role in patho-logical signaling mechanisms shared among common TD2M complications, including diabetic retinopathy (Behl et al., 2022) and diabetic kidney disease (Ansari et al., 2025; Hou et al., 2025).

When the genes annotated to the ‘Glu’ set were put together with our rSNP panel, we discovered 28 genes in the overlap. The encoded products include important enzymes, i. e. MDH2, malate dehydrogenase 2, which catalyzes a reversible NADdependent dehydrogenase reaction and glycolysis enzymes (i. e. ENO1, enolase 1, and PGP, phosphoglycolate phosphatase), as well as a number of regulatory proteins (i. e. FoxO1, USP7, SIRT6). Thus, FoxO1 has a critical role in the insulinmediated regulation of de novo glucose synthesis affecting the transcription of rate-limiting enzymes, G6Pc and Pck1 (Hall et al., 2014). Notably, multiple studies support that sirtuin 6, SIRT6, an NAD-dependent deacetylase/deacylase/mono-ADP ribosyltransferase, is involved in inhibition of FoxO1 leading to suppression of gluconeogenesis (Liu et al., 2021; Wang et al., 2023). Moreover, it was shown that the activation of deubiquitinating enzyme USP7 similarly results in a decrease in FoxO1 activity (Jiang et al., 2017).

**Fig. 4.**
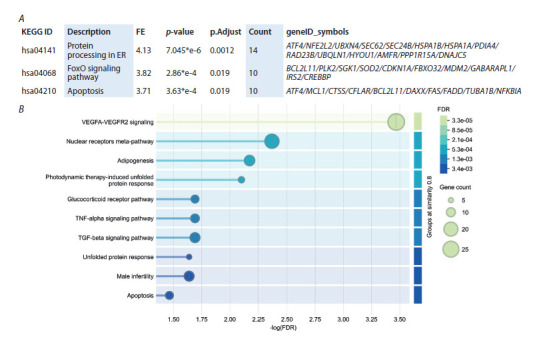
PPI network for the intersection of 28 genes as by STRING. (A) The clusters identified with K-means cluster analysis. (B) Four closely connected PPI members belonging to KEGG AMPK-signaling pathway (hsa04152). (C) A visualization of the most relevant enriched Biological Process Gene Ontology terms. Nodes of different color indicate the members of different clusters (A and B): red – cluster 1, green – cluster 2, and blue – cluster 3. The solid and the dotted lines (A) indicate connections within the same and with a different cluster respectively. Edges of different color indicate both functional and physical protein interactions (A and B): cyan – from curated databases; pink – experimentally determined; blue – gene co-occurrence; khaki – from text mining; black – coexpression; light blue – protein homology. Different enriched terms with corresponding p-values (FDR) are highlighted in different colors according to the color panel (C).

Using STRING, we constructed and analyzed a PPI network for this subset of 28 genes (Fig. 4). Three clusters were identified by the KMeans algorithm: nine protein products of cluster 1 were found to be involved in NADH metabolism and gluconeogenesis; seven from cluster 2, in regulation of gluconeogenesis, circadian rhythms and AMP-activated protein kinase activity (PRKAG2 and PRKACA); and cluster 3 was comprised of two subunits of protein phosphatase 4 complex: PPP4R3A and PPP4R3B (Fig. 4A). It is also to be noted that the protein products of four genes were functionally related to KEGG AMPK-signaling pathway (Fig. 4B). The results of functional enrichment analysis based on Gene Ontology are visualized in Figure 4C.

**Fig. 5.**
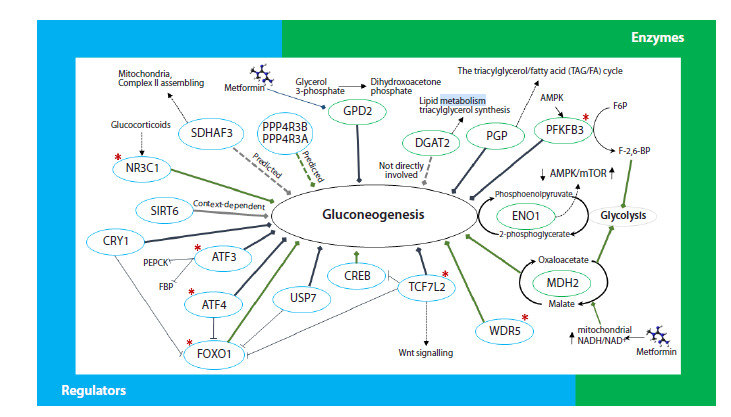
Multiple potential rSNP targets, including enzymes and regulatory proteins, known to be involved in regulation of gluconeogenesis. Blunt orange arrows indicate the activation of gluconeogenesis and deep-blue ones stand for the suppression of the process; T-shaped blue arrows indicate the suppression of transcription/translation or protein activity; red stars indicate genes from the AMPKdep gene set; AMPK, 5’ adenosine monophosphate-activated protein kinase; mTOR, mammalian target of rapamycin; Wnt, Wingless-related integration site; PEPCK, phosphoenolpyruvate carboxykinase; FBP, fructose-1,6-bisphosphatase; F6P, fructose 6-phosphate; F-2,6-BP, fructose-2,6-bisphosphate; NADH, nicotinamide adenine dinucleotide; and NAD+, the oxidized form of NAD.

For a number of these genes, the functional links with regulation of gluconeogenesis have been well described as given on the schematic representation in Figure 5. From this background we hypothesized that our rSNPs located within the corresponding promotors may be involved in regulatory signatures.

Adding the data on AMPK-dependent metformin hepatic response allows us to highlight six genes (ARPP19, ATF4, NR3C1, PFKFB3, TCF7L2, and WDR5) from a list of 28. These genes belong to the intersection of all three compared gene sets, which means the corresponding promotors harbor rSNPs; the genes were shown to be expressed in AMPK-dependent manner in hepatocytes; and the resulting proteins were involved in gluconeogenesis

Thus, NR3C1, better known as the glucocorticoid receptor (GR), has been functionally linked to stress response, immune regulation, inflammation and control of hepatic gluconeogenesis (Zhang et al., 2019). The encoded protein functions as a transcription factor enhancing the transcription of gluconeogenic genes such as PEPCK and G6Pase under fasting conditions (Beaupere et al., 2021).

ATF4, activating transcription factor 4, is a key protein in metabolic regulation involved in insulin secretion and sensitivity control and regulation of gluconeogenesis by affecting the transcriptional activity of FoxO1 (Li et al., 2022). Interestingly, a number of findings demonstrate epigenetic regulatory mechanisms targeting ATF4. For example, it was reported that microRNA (miR)-214 upregulates ATF4 expression, leading to elevated expression of FoxO1 and thus to suppression of gluconeogenesis (Zhu et al., 2018; Wang et al., 2024).

WRD5 performs multiple scaffolding functions in the context of chromatin and was reported to modulate gluconeogenic gene expression by potentiating the activity of KAT2B histone lysine acetyltransferase. Although these two genes seem to regulate a relatively small subset of gene targets in hepatocytes, both KAT2B and WDR5 are required for the expression of a large fraction of glucagon-inducible genes, indicating that they represent the important cofactors for the CREB pathway (Ravnskjaer et al., 2013).

The TCF7L2 protein is a transcription factor that plays an important role in the Wnt signaling pathway and has been implicated in blood glucose homeostasis (Ip et al., 2012) through actions even beyond pancreatic beta cells (Bailey et al., 2015).

Finally, the protein encoded by PFKFB3 is a bifunctional enzyme involved in both the synthesis and degradation of fructose-2,6-bisphosphate (F-2,6-BP), and is a potent activator of 6-phosphofructokinase-1 (PFK-1) – a trigger of aerobic oxidation for glucose metabolism. Recent studies have reported the pivotal role of PFKFB3 in the regulation of insulin resistance (Yang et al., 2023).

## Conclusion

This work identifies several novel genes and gene regulatory variants that can be activated due to metformin treatment and thus provides candidates in the human genome where nucleotide variation can lead to differences in metformin response. We conclude that metformin therapy response may depend on the identified functional SNPs within the promotors of genes for gluconeogenic enzymes and regulatory proteins.

## Conflict of interest

The authors declare no conflict of interest.
